# Removal behaviour of residual pollutants from biologically treated palm oil mill effluent by *Pennisetum purpureum* in constructed wetland

**DOI:** 10.1038/s41598-021-97789-0

**Published:** 2021-09-14

**Authors:** Farhana Aziz Ujang, Ahmad Muhaimin Roslan, Nurul Atiqah Osman, Ashreen Norman, Juferi Idris, Mohammed Abdillah Ahmad Farid, Mohd Izuan Effendi Halmi, Misri Gozan, Mohd Ali Hassan

**Affiliations:** 1 Department of Bioprocess Technology, Faculty of Biotechnology and Biomolecular Sciences, Universiti Putra Malaysia (UPM), 43400 UPM Serdang, Selangor Malaysia; 2 Faculty of Chemical Engineering, College of Engineering, Universiti Teknologi MARA (UiTM), Sarawak Branch, Samarahan Campus, 94300 Kota Samarahan, Sarawak Malaysia; 3 Department of Soil Management, Faculty of Agriculture, Universiti Putra Malaysia (UPM), 43400 UPM Serdang, Selangor Malaysia; 4 Department of Chemical Engineering, Faculty of Engineering, Universitas Indonesia, Kampus Baru UI, Depok, Jawa Barat 16424 Indonesia

**Keywords:** Environmental biotechnology, Microbiome

## Abstract

The reason for such enormous efforts in palm oil mill effluent research would be what has been singled out as one of the major sources of pollution in Malaysia, and perhaps the most costly and complex waste to manage. Palm oil mill final discharge, which is the treated effluent, will usually be discharged to nearby land or river since it has been the least costly way to dispose of. Irrefutably, the quality level of the treated effluent does not always satisfy the surface water quality in conformity to physicochemical characteristics. To work on improving the treated effluent quality, a vertical surface-flow constructed wetland system was designed with *Pennisetum purpureum* (Napier grass) planted on the wetland floor. The system effectively reduced the level of chemical oxygen demand by 62.2 ± 14.3%, total suspended solid by 88.1 ± 13.3%, ammonia by 62.3 ± 24.8%, colour by 66.6 ± 13.19%, and tannin and lignin by 57.5 ± 22.3%. Heat map depicted bacterial diversity and relative abundance in life stages from the wetland soil, whereby bacterial community associated with the pollutant removal was found to be from the families *Anaerolineaceae* and *Nitrosomonadaceae*, and phyla Cyanobacteria and Acidobacteria.

## Introduction

In Malaysia, palm oil is known as the largest agriculture sector, contributing considerably to the country's economic growth^1^. The oil palm plantation area accounts for 11% of Malaysian land^2^ and produces over 13 million tonnes of crude palm oil each year^3^. Nevertheless, huge growth entails negative externalities, especially towards the environment. Palm oil mill effluent (POME) has been identified to be one of the major sources of water pollution due to its high biochemical oxygen demand (BOD) and chemical oxygen demand (COD) concentrations^4^. As a result, POME is considered as the most costly and crucial waste to be managed, mainly due to its huge volume^5–7^. Although POME is non-hazardous, it still poses an ecological threat to the aquatic lives due to the immense oxygen consumption capacity in the photo-oxidation of its high organic compounds. Presently, palm oil mills in Malaysia are treating their POME into final discharge (POME FD) using a ponding system due to its low cost and ability to effectively reduce the generated pollutants. POME FD would typically be released to neighbouring waterways as it is the least expensive disposal practice^5^ even though it hardly met the minimum discharge limits for river quality by retaining comparatively high suspended solids (SS) and COD^8^. Table 1 shows that POME FD released still contained high amount of SS and COD value as compared to Malaysia Standard Discharge Limit. Therefore, the best way to resolve this issue is by proposing an additional polishing treatment to further reduce the contamination level prior to discharge^9^.

**Table 1:** Characteristics of palm oil mill effluent final discharge.

Parameters	Final discharge^9^	Final discharge^8^	Standard A^a^
BOD_5_ (mg/L)	65	NM	20
COD (mg/L)	520	395	80
TSS (mg/L)	217	117	50
NH_4_^+^-N (mg/L)	NM	NM	10
Colour (Pt–Co)	NM	NM	–
pH	8.50	9.3	6.0–9.0

^a^Standard Discharge Limit (Standard A), Department of Environment (DOE), Malaysia.

*NM* not measured.

To further remove the residual pollutants from POME FD, the use of green plants along with proper soil amendment such as phytoremediation in a constructed wetland (CW) can be applied. Pollutants are innately degraded, removed or immobilised during phytoremediation, where the process costs way less, and with low carbon footprint as compared to other techniques, e.g. adsorbent, bioreactors, or convective drying, in treating a sizeable volume of POME^10,11^. As an engineered wetland, CW can be designed to mimic the natural wetlands to treat wastewater^12^. Vegetation on the CW will induce the growth of soil microorganisms, and foster an ecosystem surrounding the roots, primarily to remove and immobilise contaminants from the wastewater. The resulted wastewater with lesser residual pollutants is important, because it will have lower impact to the receiving water body, as compared to wastewater without CW. Several plants have been studied to effectively remove pollutants from the environment through several methods of phytoremediation such as rhizodegradation, phytoimmobilisation, phytoextraction, phytodegradation, phytofiltration, phytostabilisation, phytodesalination, and phytovolatilisation^13^. *Pennisetum purpureum* (Napier grass) is one of the plant species used for the remediation of industrial and agricultural effluents due to its rapid growth rate and ability to survive in highly contaminated soil. A study has also reported that *P. purpureum* has the potential to absorb significant amount of excess nutrients in wastewater^14^. Furthermore, soil microorganisms in CW have also been proven to associate in pollutant removal such as nitrogen^15^, organic matters^16^, industrial organic pollutants^17^, and emerging organic contaminants^18,19^, either as free-floating microorganisms in interstitial water, as well as bound to CW substrates and plant roots^20^.

Since the POME FD contains a lot of residual organic pollutants, which can become nutrients for plants such as *P. purpureum* growth, it is important to understand the removal behaviour of these residual pollutants in CW, and the shifting of microorganism community associated with the process. Although previous studies have analysed independent water samples^21,22^ as well as substrate samples^23,24^ to understand the microbial characteristics in CW, the study on removal behaviour of residual pollutants in POME FD using CW is still lacking. The objective of this study is to understand the removal behaviour of pollutants from POME FD in a CW using *P. purpureum* to achieve the receiving river water quality. A bacterial community study was also conducted to further understand the relationship between the microorganisms and pollutants removal.

## Materials and methods

### Experimental designs

**Figure 1:**
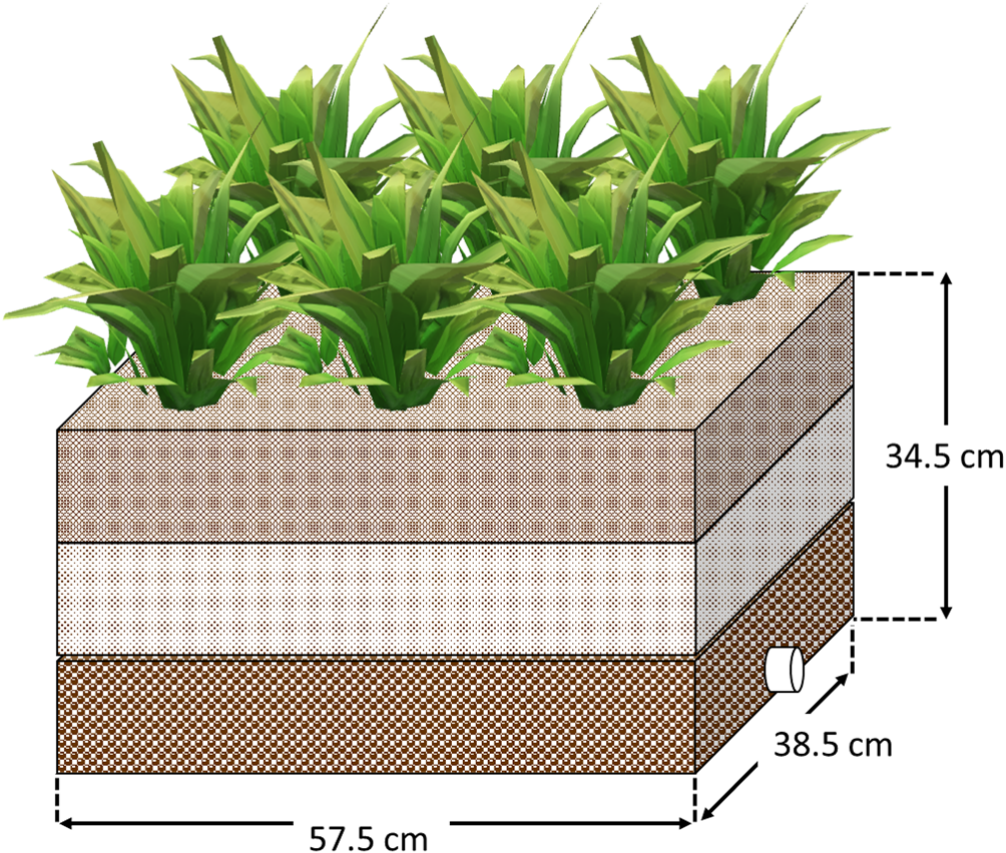
Design of the constructed wetland system.

The constructed wetland system was set up according to Fig. 1 in triplicate for every study, in a greenhouse. Every tank used to create the wetland has a working capacity of 60 L, measuring approximately 40.5 cm high, 57.5 cm long, and 38.5 cm wide. The tanks were filled with three types of media or substrate for plant cultivation and wastewater treatment. From the bottom, 11.5 cm height of stones with 3–5 cm diameter was lined as the first layer. The second layer was filled with coarse sands with 11.5 cm height, while the third layer was 11.5 cm height of fine sands. Each medium has its function; stone or gravel in the bottom layer serves as the supporting layer, coarse sand in the second layer is the main substrate layer, and the fine sand in the upper layer facilitates the dispersion of wastewater and growth of plants. At the bottom of the CW, and outlet pipe (diameter 2.5 cm) is installed to collect the effluent sample. *P. purpureum* (common cultivar) used in this study was obtained with permission from Biomass Technology Laboratory, Universiti Putra Malaysia, and was used and maintained within the facility. It was grown for 2 months prior to transplanting into three wetland tanks that were designated for this experiment.

### Experimental procedure

The sample of POME FD used in this study was collected from a palm oil mill located in Felda Pasoh, Negeri Sembilan. The FD was retrieved from the final processing line of the POME treatment system within the POME biopolishing plant, and immediately collected as the effluent flew into the drain. The effluent samples were then kept at 4 °C before use.

The wetlands were supplemented with 5 L of POME FD on daily basis. The effluent from each tank was channelled through a drainpipe (2.5 cm diameter) at the bottom of the tank, which was then collected for analysis purposes. The sampling was done periodically every three days from each tank for 75 days of treatment. Following sampling, the influent and effluent samples from the CW were then immediately analysed for COD, TSS, ammonium nitrogen (NH_4_^+^-N), colour, and tannin and lignin, according to Standard Methods for the Examination of Water and Wastewater^25^. These samples were also analysed for heavy metals by inductively coupled plasma mass spectrometry (ICP-MS).

### Plant physiology

The measurement for *P. purpureum* growth was performed once per week. The measurement was also taken before the experiment, and on the last day of treatment.

### Microbial analysis

Sediments from three different points in each CW were collected before combined into a single composite sample. The sediments and the mixed liquors were extracted with a DNA extraction kit from MOBIO PowerSoil to recover complete genomic DNA, and preserved at – 20 °C. The sample was then proceeded with a high-throughput MiSeq Illumina sequencing platform to analyse the influence of the POME FD and wetland effluent on the bacterial community composition. The preserved DNA was sent to Biolution Resources, Ampang, Selangor, Malaysia, where the sequencing was conducted on the 16S rRNA gene in the V3 and V4 regions. The taxonomy of each 16S rRNA gene sequence was analysed by RDP Classifier against the Silva (SSU123) 16S rRNA database using a confidence threshold of 0.7. The number of all OTU in rarefaction curve was in the saturation phase, indicating that OUTs have been sufficiently recovered in the sequencing. The result from Biolution Resources were then imported and analysed in Microsoft Excel spreadsheet with pivot tables. This table was analysed to create a heatmap using the relative abundance of each genus. The colours on the heatmap represent the relative abundance. For each row, the rule applied identified the highest (red) and the lowest (green) values, and assigned colour gradient for the values in between.

## Results and discussion

### Removal of COD in constructed wetland

**Figure 2:**
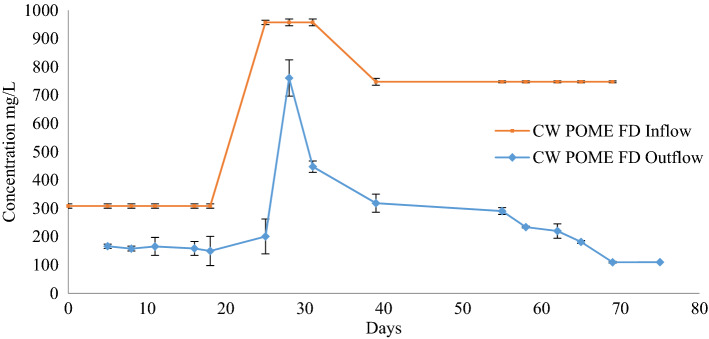
The chemical oxygen demand (COD) reduction profile after the treatment using constructed wetland system for 75 days.

The CW had a steady state of outflow values after 75 days of operation, and good plant growth was observed. The system started to release effluent on the third day after the first influent was added. COD in the effluent was in adaptation process in the beginning because microorganisms and plants had not adapted and matured in the conditions provided by the FD^26^. Generally, the COD removal efficiencies in wetlands were 88.4 ± 0.24% at day 75. COD concentration gradually decreased with time from day 27 to 75 as shown in Fig. 2. COD concentration showed a continuous reduction as compared to the input. Although a higher COD concentration of POME FD was supplied at day 24, the CW managed to reduce it by 15.84 ± 0.22% at day 28.

The concentration of influent had an effect on the elimination of COD. The removal of COD can be explained by the function of the *P. purpureum* in the CW as the plant roots exuded additional organic matters^27^, which led to the changes of COD concentration. After 27 days, the living roots had flourished and became sturdier, which had stimulatory effect on soil organic matter decomposition, thus indicating development of roots-induced microbiota^28,29^. On top of that, the microbial communities within the CW ecosystem were seeded by the presence of bacteria in the POME FD that were involved in anaerobic digestion. One of the major bacterial constituents that can be found in the POME FD was *Pseudomonas* sp. This bacterium is capable of dye degradation or colour removal, biodegradation of hydrocarbon, and insecticide degradation such as Malathion^30,31^. It has been reported that *Pseudomonas aeruginosa* is capable to reduce the COD level from POME in a microbial fuel cell^32^, as well as the COD level of urban wastewater^33^.

### Removal of total suspended solid in constructed wetland

**Figure 3:**
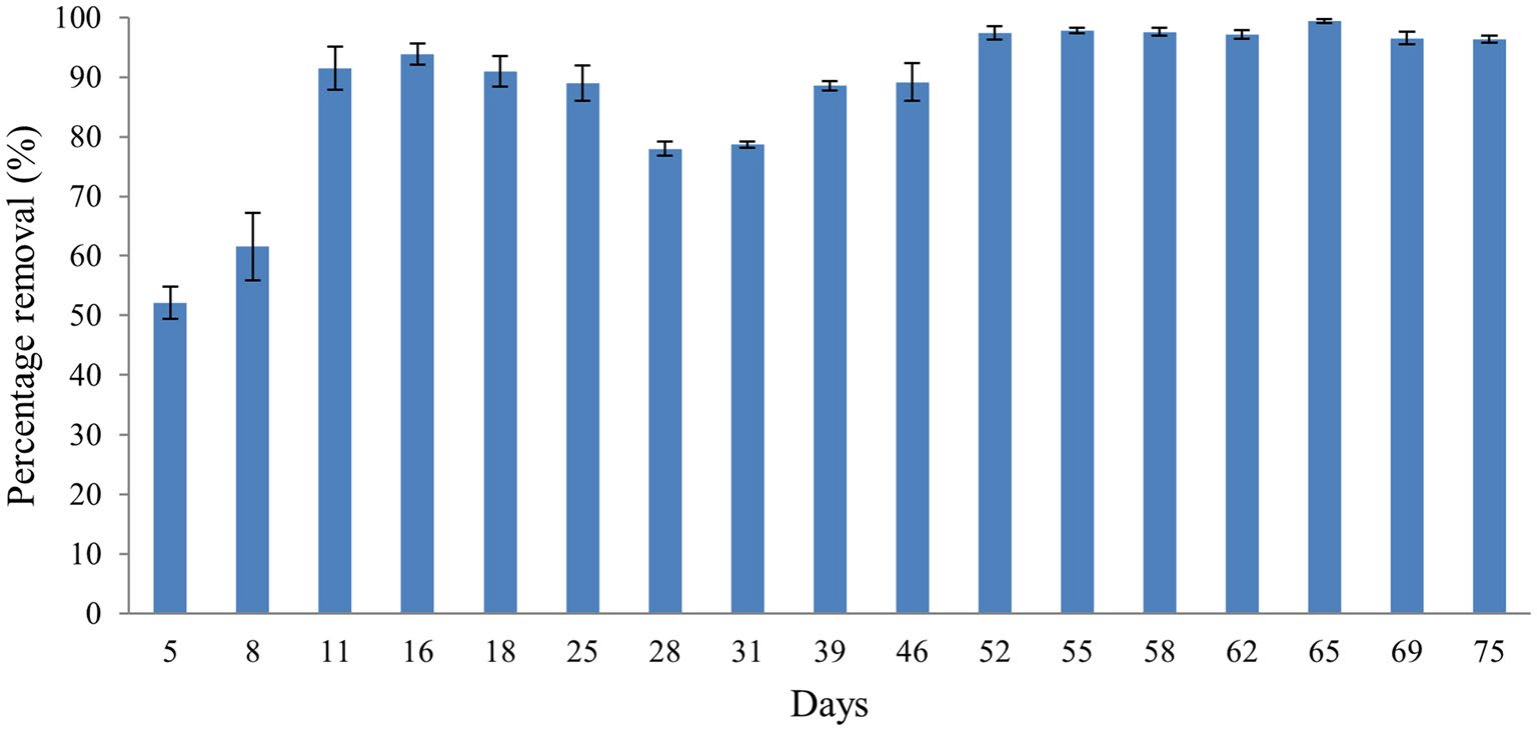
The total suspended solids (TSS) reduction profile after the treatment using constructed wetland system for 75 days.

Figure 3 shows the removal of TSS from POME FD. Different concentrations of POME FD were supplied throughout this study to simulate a real palm oil mill. Even though the wastewater input into the wetland did not have the same initial TSS concentration, the system still managed to reduce the TSS up to 88.1 ± 13.3%. The POME FD concentrations supplied were 129 ± 3.2 mg/L (day 0–24), 295 ± 4.3 mg/L (day 27–39) and 553.7 ± 11.2 mg/L (day 46–72). The concentrations of TSS from the CW outlet for day 25, day 46, and day 75 were 13.6 ± 3.6 mg/L, 30.2 ± 8.5 mg/L, and 19.4 ± 3.3 mg/L, respectively.

**Figure 4:**
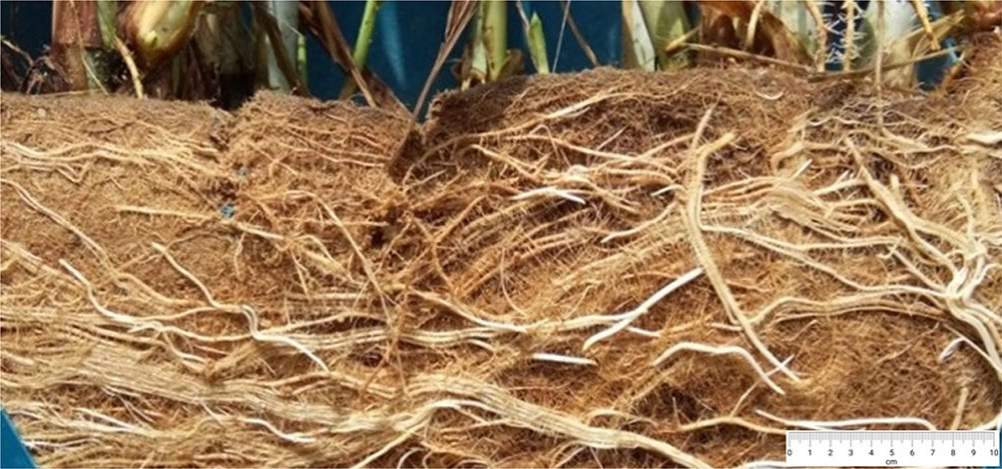
The root of *P. purpureum* in the constructed wetland at day 75.

Dhulap and Patil^34^ have shown the potential of *P. purpureum* to minimise TSS in sewage by 55.17%. The removal strategies for TSS in CW are via sedimentation and filtration. Particulates were removed whenever the POME FD seeped through the developed root masses of *P. purpureum*, gravel, and sand. This finding matches current finding as the sedimentation of the solids happened at the top of the CW, and the roots of *P. purpureum* also showed massive growth in the entire CW, as shown in Fig. 4. Typically, the CW has a lengthy hydraulic retention time, often several days, to extract settle-able and floatable solids while bacterial growth and interactions with adsorbed species remove non-settling solids.

### Removal of tannin and lignin in constructed wetland

Tannins, also known as polyphenols, are by far the most abundant macromolecules contained in POME that greatly affect its colour, and create musty odour^35^. The tannins leaching onto the run-off wastewater from the palm oil processing give the brown colouring effect to the POME. From the results obtained, tannin and lignin concentrations were decreasing in three phases as the inlet of the CW was taken in different batches for three consecutive months at Felda Pasoh, Negeri Sembilan.

**Figure 5:**
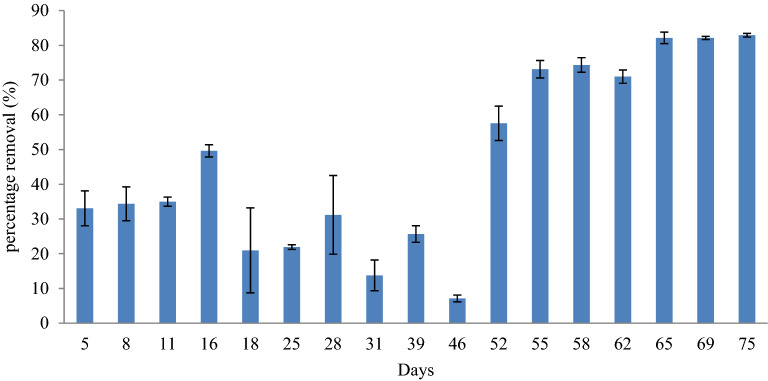
The tannin and lignin reduction profile after the treatment using constructed wetland system for 75 days.

Overall, the tannin and lignin contents decreased from the initial concentration as shown in Fig. 5. The concentrations of tannin and lignin in the inlet of CW were 8.7 ± 0.6 mg/L, 14.5 ± 0.2 mg/L, and 21.8 ± 0.4 mg/L. The removal of tannin and lignin was showing as early as day 3, which then reduced to 6.4 ± 0.9 mg/L and 4.5 ± 0.6 mg/L at day 15. The concentration drastically increased on day 27 due to the high concentration of the inlet of CW. Overall, the removal of tannin and lignin was 57.5 ± 22.3% using this CW, and believed to be related to the loading rates and hydraulic retention time (HRT). According to Grismer and Sherpherd^36^, a smaller loading rate and greater HRT can influence the removal efficiency to reach nearly 100% provided that the recirculation system of effluent is applied. It was reported that CW have been proven to have the ability to reduce the concentration of tannin and lignin in wood waste leachate^37^. The study reported that there was a total reduction of 42% of tannin and lignin using the CW planted with a cattail (*Typha* sp.). This is in agreement with Grismer and Sherpherd^36^ who reported successful reduction of up to 77.9% of tannin and lignin from the winery process wastewater by subsurface flow wetland. Comparing both studies with this study, better removal of tannin and lignin was achieved on day 52 to day 75 of 82.9 ± 0.47%, which is higher than reported literature^36,37^.

### Removal of effluent colour in constructed wetland

The colour of wastewater is one of the parameters that must be treated. A high concentration of colour in wastewater has an impact on the environment by reducing the penetration of sunlight into the water body, and this is often caused by organic matters which include suspended and dissolved particles. Dissolved organic matters, such as humus, peat, and plant decay contribute to the yellow or brown colour of the wastewater, whereas as the dissolved organic acids including tannin and lignin give the wastewater a tea colour. According to Mohammed and Chong^35^, there is a correlation between TSS, and tannin and lignin contents of POME, and the resulting effluent colour.

**Figure 6:**
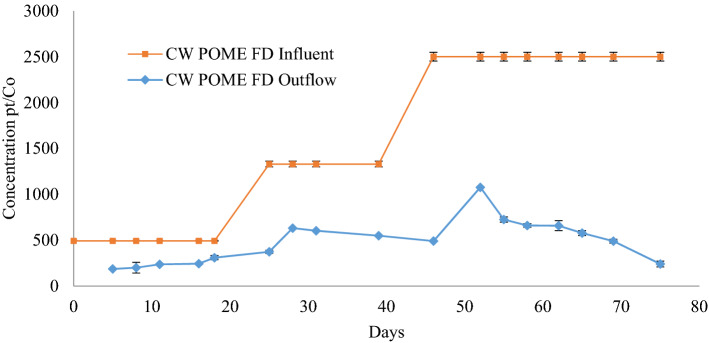
The colour reduction profile after the treatment using constructed wetland system for 75 days.

By reducing the concentration of TSS, and tannin and lignin in the POME FD, the colour concentration may also decrease. In this study, the colour started to reduce from day 3 to day 75. The colour concentration in this study is represented by platinum-cobalt (Pt–Co). Initially, the colour of POME FD recorded was 493 ± 3 Pt–Co (day 0–24), 1330 ± 31 Pt–Co (day 27–39), and 2501 ± 47 Pt–Co (day 46–75). Based on Fig. 6, the colour of treated POME FD reduced to 310 ± 16 Pt–Co (day 24), to 491 ± 16 Pt–Co (day 45), and finally to 238 ± 4 Pt–Co (day 75).

Overall, the CW managed to yield 57.5 ± 22.3% of colour removal, thus indicating the effectiveness of using *P. purpureum* in removing the colour^34^. It is expected that the dissolved solids that add to the colour will be washed out by the CW. The presence of bacteria in the CW often also leads to a decrease in colour quality^32^.

### Removal of ammonium nitrogen in constructed wetland

**Figure 7:**
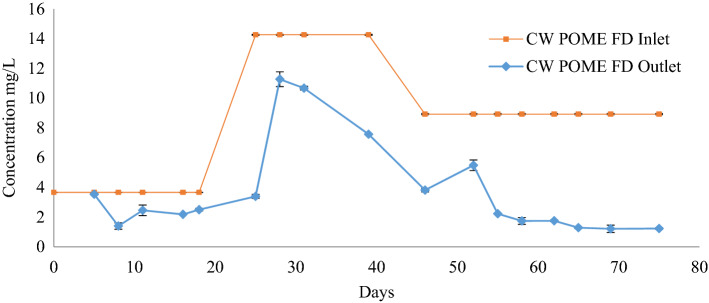
The ammonia reduction profile after the treatment using constructed wetland system for 75 days.

As the concentration of ammonium nitrogen (NH_4_^+^-N) was high inside the POME FD (14.26 ± 0.04 mg/L), the CW was used to reduce its concentration. Figure 7 exhibits the ammonia removal after 75 days in the CW. The influents of the CW were different at 3.66 ± 0.01 mg/L, 14.26 ± 0.04 mg/L, and 8.92 ± 0.04 mg/L according to the date of sampling for every batch which was in July, August, and September, respectively. An extended HRT would lengthen the contact interval between microorganisms and organic matters in wastewater, thus increasing the efficiency of NH_4_^+^-N, contrary to a shortened HRT which would inadequately remove the ammonia as a result of reduced time for nutrient uptake by the nitrifying bacteria, thereby slowing down the proliferation of autotrophic bacteria^38^.

Even though the initial concentration had higher values, at the end of the treatment, the CW managed to reduce the concentration of NH_4_^+^-N to 1.4 ± 0.4 mg/L, 3.8 ± 0.4 mg/L, and 1.2 ± 0.1 mg/L, of about 64.8 ± 9.6%, 56.6 ± 4.6%, and 86.2 ± 1.7%, respectively. The NH_4_^+^-N concentrations were only fractionally declined within the first few days by 7.3 ± 0.4% due to a large amount of dissolved oxygen that has been consumed by the organic matters for degradation. NH_4_^+^-N concentrations stabilised after 57 days, at 1.7 ± 0.2 mg/L, and on day 60 at 1.74 ± 0.04 mg/L. On days 63, 66, and 75, there were stable NH_4_^+^-N concentrations at 1.3 ± 0.3 mg/L, 1.2 ± 0.1 mg/L, and 1.2 ± 0.1 mg/L, respectively. This suggests that there has been a combination of NH_4_^+^-N sorption onto CW substrates, microbial assimilation, and nitrification at the air–water interface^39^. NH_4_^+^-N concentrations steadily declined as HRT increased, with removal performance recorded at 74.4 ± 0.4% and 84.2 ± 1.8%.

A study has reported the low reduction trend of hydrocarbon in the first 60 days, and greatly decreased towards the end of the experiment once the maturity phase has been achieved^40^. This explains that the removal of NH_4_^+^-N did not only rely on the adsorption by the CW substrates, but also on photosynthesis acclamation and root development of the plants, as well as sufficiency of oxygen supply to rhizosphere aerobic microorganisms^13^. In context of biogeochemical nitrogen cycle, there are three oxidative steps involved in converting ammonia to nitrate (NH_4_^+^  → NH_2_OH → NO_2_^−^  → NO_3_^−^)^41^. *P. purpureum* will absorb the nitrates and enhance its growth. In this study, the CW managed to reduce the concentration of NH_4_^+^-N in POME FD by 62.3 ± 24.8%.

### Effluent characteristics in the constructed wetland

Overall, the effluent characteristics after being treated in the CW showed a higher reduction in FD concentration as compared to its initial concentration. Table 2 shows the different initial concentrations of FD in July, August, and September due to the different seasons. In July, the concentration of FD was quite low as compared to the concentrations in August and September since it was a rainy season. Thus, the concentration of the FD was diluted by the rain. On the contrary, August and September were dry seasons, hence the concentrations of FD were quite high. However, even with different initial concentrations, 52.4 ± 8.2%, 66.9 ± 2.2%, and 85.2 ± 0.5% removal of COD was recorded in each three different months. Concentrations of TSS were reduced to 88.9 ± 2.9%, 90.5 ± 2.7%, and 96.4 ± 0.6% in those months, respectively. For the concentration of colour, September showed the highest removal (90.4 ± 0.18%), followed by 63.1 ± 0.36% in August. and 50.4 ± 4.57% in July. The ammonia concentration also showed an increment in removal in July, August, and September by 31.1 ± 3.8%, 72.9 ± 3.4%, and 86.2 ± 1.7%, respectively.

**Table 2:** The values of initial concentrations of wetland influent and the final concentrations of wetland effluent with the removal percentage in 3 months of continuous treatment process.

Parameter	July (1st month)			August (2nd month)			September (3rd month)		
	Initial conc.	Final conc.	Removal (%)	Initial conc.	Final conc.	Removal (%)	Initial conc.	Final conc.	Removal (%)
COD (mg/L)	308.5 ± 7.7	149.6 ± 24.6	52.4 ± 8.2	957.3 ± 12.1	318.6 ± 20.0	66.9 ± 2.2	747.3 ± 3.2	109.8 ± 3.1	85.2 ± 0.5
TSS ﻿(mg/L)﻿	129.4 ± 3.2	13.6 ± 3.6	88.9 ± 2.9	295.0 ± 4.3	30.2 ± 8.5	90.5 ± 2.7	553.7 ± 11.2	19.4 ± 3.3	96.4 ± 0.6
Tannin and lignin (mg/L)	8.7 ± 0.6	4.5 ± 0.6	50.7 ± 2.6	14.5 ± 0.2	9.2 ± 1.6	25.3 ± 2.1	21.8 ± 0.4	3.7 ± 0.1	83.1 ± 0.5
Colour (Pt/Co)	493 ± 3.5	244.5 ± 22.5	50.4 ± 4.6	1330.8 ± 31.5	491.3 ± 4.8	63.1 ± 0.4	2501 ± 47.8	238.3 ± 4.5	90.4 ± 0.2
NH_4_^+^-N (mg/L)	3.7 ± 0.01	2.5 ± 0.1	31.1 ± 3.8	14.3 ± 0.04	3.8 ± 0.4	72.9 ± 3.4	8.9 ± 0.04	1.2 ± 0.1	86.2 ± 1.7

Overall, the CW managed to reduce the concentration of COD, TSS, tannin and lignin, colour, and ammonia in the FD. Approximately 62.2 ± 14.3%, 88.1 ± 13.3%, 57.5 ± 22.3%, 66.6 ± 13.2%, and 62.3 ± 24.8% of COD, TSS, tannin and lignin, colour, and ammonia were reduced from the effluent sample, as summarised in Table 2. All parameters met the river water quality except for COD. For future consideration, this study suggests an extended HRT to achieve a better efficacy in reducing the COD.

The designed CW treatment using *P. purpureum* showed a comparable removal with other studies, while some of the parameters showed better removal as shown in Table 3. A comparable COD removal efficiency of 66.2% was achieved, which was comparable to 66.7%^43^ and 65.9%^44^. For NH_4_^+^-N removal, this study recorded 62.3% removal from POME FD, which is within the range of other studies (65.9%^48^ and 67.2%^42^). Interestingly, the TSS showed a greater removal (85%) as compared to a previous study (68%)^43^.

**Table 3:** The comparison of removal of pollutants with other studies.

COD (%)	TSS (%)	Tannin and lignin (%)	Colour (%)	NH_4_^+^-N (%)	Types of wetland	Types of wastewater	Types of plant	Refs.
56.2	–	–	–	67.2	HSF-CW^a^	Modified river water	Common reeds	^42^
64.0	68.0	–	–	–	VSF-CW^b^	Secondary sewage	*P. purpureum*	^14^
66.7	–	–	84.5	–	HSF-CW	Domestic sewage	Reed grass	^43^
65.9	–	–	–	30.5	HSF-CW	Wastewater from livestock	*Potamogeton crispus*	^44^
49.72	–	–	–	42.8	VF-CW^c^	Secondary wastewater	Sweet flag	^45^
42.0	85.0	–	–	39.0	HSF-CW	Domestic wastewater	*P. purpureum*	^46^
41.3	–	–	–	65.9	HSF-CW	Secondary Wastewater	*Potamogeton crispus*	^47^
66.2	88.1	57.5	66.6	62.3	VSF-CW	POME FD	*P. purpureum*	This study

^a^Horizontal surface flow constructed wetland.

^b^Vertical surface flow constructed wetland.

^c^Vertical flow constructed wetland.

### Removal of trace elements in constructed wetland

The POME FD could be used as a source for nitrogen, phosphorus, potassium, magnesium, and calcium for plant growth^48^. The elements that were present in the POME FD are listed in Table 4. In phytoremediation studies, grass is the most common plant that has been used as it has high tolerance towards heavy metals. For this study, *P. purpureum* was used in the CW owing to its extensive fibrous roots (Fig. 4) that could reduce leaching and erosion^49^. All these characteristics give *P. purpureum* phytoremediation advantage where it can help to reduce the trace elements inside the POME FD. The phytoremediation concept managed to reduce the concentrations of potassium, silica, caesium, rubidium, sodium, copper, magnesium, calcium, strontium, and manganese in POME FD by 82.19%, 80.51%, 71.17%, 66.07%, 58.46%, 59.27%, 56.81%, 35.80%, 35.57%, and 20.81%, respectively, as shown in Table 4.

**Table 4:** The trace element concentrations in influent and effluent for wetland treatment.

Trace element	Influent (ppm)	Effluent (ppm)	Removal (%)	Standard discharge limit by DOE (ppm)
Potassium	201.81 ± 3.02	35.94 ± 1.01	82.19	–
Silica	25.51 ± 1.05	4.97 ± 0.14	80.51	–
Caesium	0.111 ± 0.001	0.032 ± 0.002	71.17	–
Rubidium	4.76 ± 0.032	1.62 ± 0.12	66.07	–
Sodium	14.38 ± 0.45	5.97 ± 0.63	58.46	–
Copper	0.033 ± 0.0003	0.013 ± 0.001	59.27	0.2
Magnesium	318.25 ± 5.01	137.45 ± 1.02	56.81	–
Calcium	70.32 ± 0.87	45.15 ± 2.87	35.80	–
Strontium	0.30 ± 0.01	0.19 ± 0.01	35.57	–
Manganese	0.042 ± 0.001	0.033 ± 0.001	20.81	0.2

The concentration of silica in POME FD was quite high (25.51 ± 1.05 ppm) before treatment. However, after treatment with CW, the concentration of silica was reduced by 80.51%. This result shows the capability of *P. purpureum* to remediate silica by absorbing and immobilising it. Silica will be uptaken by the plant, and processed to form phytoliths or silica bodies which infill the cell walls and lumina of certain plant cells^50^. The uptake of silica by the plant improves its quality by showing increased chlorophyll content and plant growth^51^. On the other hand, the plant in the CW also shows a rapid growth in “Plant physiology” section. Based on this, it can be concluded that the silica uptake by the plant managed to increase the chlorophyll content and growth of *P. purpureum* while at the same time reduced the concentration of silica inside the POME FD. It has been proven that plants are capable to reduce and remediate caesium by 59.3% through accumulation at the dry shoots, which is similarly shown in this study with caesium removal of 71.17% by *P. purpureum* in the CW^52^.

### Plant physiology

**Figure 8:**
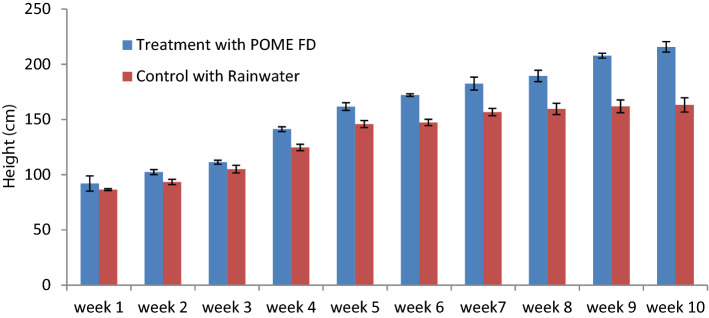
The growth of *P. purpureum* in the constructed wetland system for 75 days.

The growth performance of *P. purpureum* is measured in term of height, of as shown in Fig. 8. The initial heights of *P. purpureum* were 92 cm and 86 cm for treatment with POME FD and control with rainwater, respectively. On the last day of experiment, the height of *P. purpureum* treated with POME FD increased by 135 ± 12%, but only 88 ± 9% in control. This is due to the uptake of nutrient from the water sources^53^. A study^54^ reported that plant height increment of 61 ± 2% can be observed when it was treated with POME FD. As mentioned in "Removal of trace elements in constructed wetland" section, silica increases the growth of the plant, while potassium functions as the major osmoticum of the plant cells which controls cellular expansion^53^.

### Bacterial community analysis

**Figure 9:**
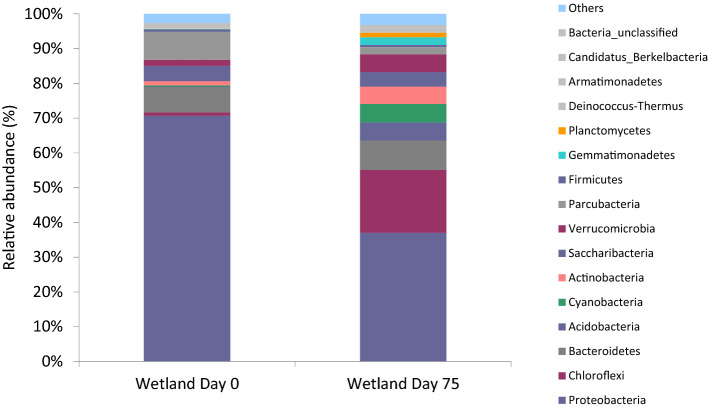
Relative abundance of the dominant phylogenetic groups at each sampling point during this study with relative abundance at the phyla level with > 0.002%.

The other component of wetlands such as microorganisms have been proven to help on the reduction of the pollutants or contaminants in a CW, such as ammonium, nitrate, organic matters, industrial organics, and emerging organic contaminants/pollutants^15–18^. The submerged plant litter did affect the soil properties and microbial diversities in a CW^55^. As shown in Fig. 9, Proteobacteria yielded the highest percentage in the phylum, ranging from 37 to 70.7%. Proteobacteria is one of the three most abundant soil bacteria phyla, and the most widespread and ubiquitous. These phyla are found dominant in wastewater treatment^23,56^. It was reported that Proteobacteria had a high resistance towards heavy metals^57^. The Proteobacteria and Firmicutes phyla can live together in harsh environments, and have been shown to have the potential to remove a broad-spectrum of heavy metals^58^. Proteobacteria are copiotrophic and root-associated bacteria that influence NO_3_^−^-N and NH_4_^+^-N in the soil^45^. Proteobacteria do not show a consistent change in depth^59,60^. Proteobacteria including *Nitrosomonas*, *Nitrosococcus*, and *Nitrosospira* have been reported as ammonia-oxidising bacteria^61^.

Chloroflexi is a phylum of primarily gliding, filamentous bacteria possessing a wide diversity of metabolisms and ecological roles, but are best known as photoheterotrophs^62^. Chloroflexi percentage at the beginning of the system was low (0.96%) but increased thereafter to 18.11%. Wetland soils are characterised by high soil moisture, and this habitat may be suitable for some bacterial phyla such as Chloroflexi to grow^63^. Since it is photoheterotrophic, it cannot use CO_2_ as a carbon source, but use the organic compounds instead, thus helping in the removal of pollutants. Chloroflexi are aerobic and anaerobic thermophiles, filamentous anoxygenic phototrophs, and anaerobic organohalides^64^. Since this phylum is anaerobic, it can survive in the CW even though the system does not have aeration.

Acidobacteria in this study also increased from 0.14 to 5.2% which shows the capability of these phyla to survive and adapt in the CW condition. These phyla have a unique ability including the ability to use nitrite as a nitrogen source, which gives positive impacts to soil macro- and micronutrients, and these phyla can also express multiple active transporters.

Cyanobacteria are phyla that contain members that possess two photosystems that are coupled in series to perform oxygenic photosynthesis. Cyanobacteria occupy a privileged position among microorganisms because of their role in carbon and nitrogen cycles^65^. These phyla also showed an increment in abundancy in the CW after treatment for 102 days. The phyla increased from 0.39 to 5.29%, which gives a clear indication that Cyanobacteria did involve in the pollutant reduction.

**Figure 10:**
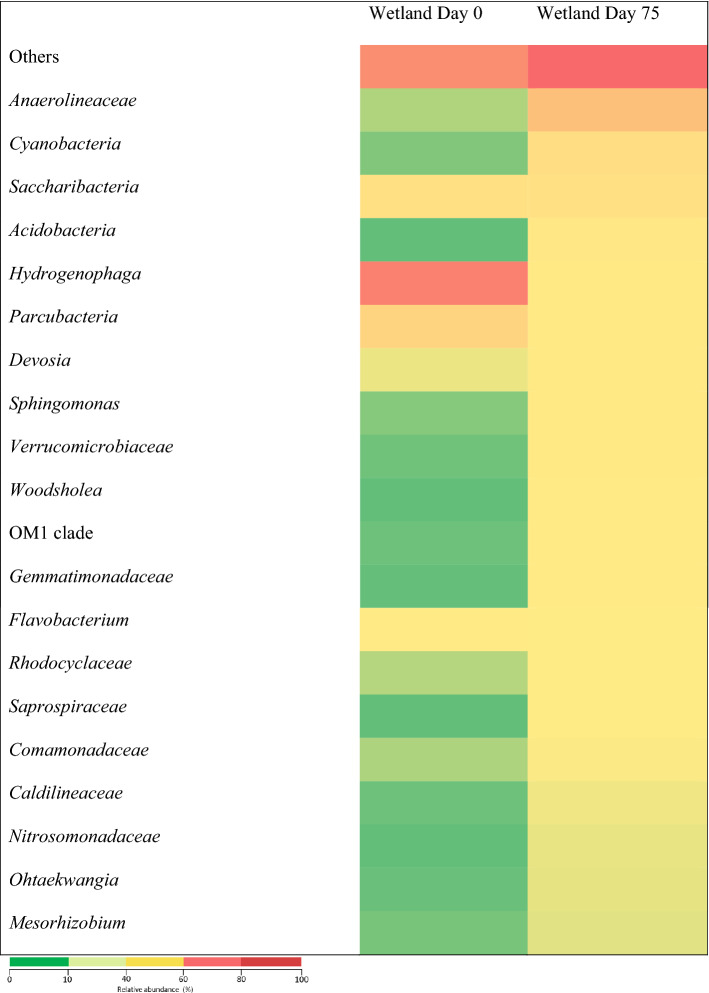
Heatmap of the relative abundance of genera and orders in the POME FD (day 0) and the treated POME FD (day 102).

Based on Fig. 10, *Anaerolineaceae*, *Cyanobacteria*, *Acidobacteria*, and *Nitrosomonadaceae* were detected in the samples of POME FD and treated POME FD. These four genera showed an increment in the CW after the treatment of POME FD for 75 days. Given the fermentative metabolism-capability to consume carbohydrates and proteinaceous carbon sources under anaerobic conditions, *Anaerolineaceae* growth had increased from 0.67 to 13.21%, signaling its participation in COD and ammonia removals^66^.

*Cyanobacteria* also showed an increment in the CW up to 93.9%. In the beginning, it was only 0.3%, but at the end of the system, it increased to 4.92%. This also shows the effectiveness of this genus to grow in this condition, and aid in the removal of pollutants in the POME FD. *Cyanobacteria* may affect the abundance of several genera across several phyla, in which most are associated with nitrogen metabolism. For example, the increase in the abundance of *Cyanobacteria* under nitrogen addition may be due to its great demand for nitrogen. *Cyanobacteria* cells are composed of nitrogen, nitrate and ammonium, which are common sources of nitrogen for *Cyanobacteria*. *Cyanobacteria*are facultative bacteria.

At the beginning of the experiment, *Acidobacteria* only showed 0.06% abundance, but after 102 days, the value increased to 2.44%. This also shows the ability of this genus to grow in the CW, which suggest its association in the removal of pollutants in the POME FD. Meanwhile, *Nitrosomonadaceae*, which belongs to *β-proteobacteria*, is also an ammonium-oxidising bacteria involved in the nitrification process^67^. This genus’ increment from 0.07 to 1.1% provides strong proof that nitrification had occurred in the CW which led to the decrement of ammonia nitrogen and total nitrogen inside the POME FD.

## Conclusions

1. The proposed system had successfully produced effluent that met the river quality standard for all parameters, except for COD. In this study, COD removal was 62.2%, TSS by 88.1%, tannin and lignin by 57.5%, colour by 66.6%, and ammonia nitrogen by 62.3%.
2. The trace elements were substantially reduced; silica by 80.5%, caesium by 71.2%, rubidium by 66.1%, strontium by 35.6%, magnesium by 56.8%, manganese by 20.8%, and copper by 59.3%.
3. The cultivated *P. purpureum* showed higher growth rate as compared to control by 16.4% with nutrients (macro-nutrients) uptake efficiency from the POME FD achieved at 54.6% for nitrogen, 91.8% for phosphorus, 75% for potassium, and 58.5% for sodium.
4. It is suggested that *Anaerolineaceae*, *Cyanobacteria*, *Acidobacteria*, and *Nitrosomonadaceae* were associated with the residual pollutant removal.
5. This study proposed a promising low-cost system that has remarkable implications for the quality of the treated effluent.
